# The diversified role of mitochondria in ferroptosis in cancer

**DOI:** 10.1038/s41419-023-06045-y

**Published:** 2023-08-14

**Authors:** Yu’e Liu, Shiping Lu, Lei-lei Wu, Liang Yang, Lixue Yang, Jinghan Wang

**Affiliations:** 1 Institute of Hepatobiliary and Pancreatic Surgery, Department of Hepatobiliary and Pancreatic Surgery, Shanghai East Hospital, School of Medicine, Tongji University, Shanghai, 200120 China; 2 Center for Translational Research in infection and Inflammation, School of Medicine, Tulane University, New Orleans, LA 70112 USA; 3 Department of Thoracic Surgery, Shanghai Pulmonary Hospital, School of Medicine, Tongji University, 200433 Shanghai, China; 4 Department of Hepatobiliary Surgery, The First Affiliated Hospital of Ningbo University, Ningbo, 315010 China; 5 Department of Biliary Tract Surgery II, Eastern Hepatobiliary Surgery Hospital, Shanghai, 200438 China

**Keywords:** Cell death, Oncogenesis

## Abstract

Ferroptosis is a form of regulated cell death induced by iron-dependent lipid peroxidation, and it has been studied extensively since its discovery in 2012. Induced by iron overload and ROS accumulation, ferroptosis is modulated by various cellular metabolic and signaling pathways. The GSH-GPX4 pathway, the FSP1-CoQ10 pathway, the GCH1-BH4 pathway, the DHODH-CoQH2 system and the sex hormones suppress ferroptosis. Mitochondrial iron metabolism regulates ferroptosis and mitochondria also undergo a morphological change during ferroptosis, these changes include increased membrane density and reduced mitochondrial cristae. Moreover, mitochondrial energy metabolism changes during ferroptosis, the increased oxidative phosphorylation and ATP production rates lead to a decrease in the glycolysis rate. In addition, excessive oxidative stress induces irreversible damage to mitochondria, diminishing organelle integrity. ROS production, mitochondrial membrane potential, mitochondrial fusion and fission, and mitophagy also function in ferroptosis. Notably, some ferroptosis inhibitors target mitochondria. Ferroptosis is a major mechanism for cell death associated with the progression of cancer. Metastasis-prone or metastatic cancer cells are more susceptible to ferroptosis. Inducing ferroptosis in tumor cells shows very promising potential for treating drug-resistant cancers. In this review, we present a brief retrospect of the discovery and the characteristics of ferroptosis, then we discuss the regulation of ferroptosis and highlight the unique role played by mitochondria in the ferroptosis of cancer cells. Furthermore, we explain how ferroptosis functions as a double-edged sword as well as novel therapies aimed at selectively manipulating cell death for cancer eradication.

## Facts

- Ferroptosis is a programmed cell death characterized by excessive intracellular iron ions and accumulated lethal lipid-based ROS, and it is regulated by various metabolic pathways.
- Mitochondria play a diversified role in the process of ferroptosis, the iron metabolism, the OXPHOS pathway, ROS, etc. are highly involved in ferroptosis.
- Will cancer cells have different ferroptosis tendencies if they have different energy resources, either relying more on mitochondrial respiration or glycolysis?

## Open questions

- How to selectively target cancer cells’ mitochondria to induce their ferroptosis?
- How can normal cells that are undergoing ferroptosis be rescued by mitochondria reprogramming?
- Does mitochondrial metabolism reprogramming function in simultaneous conjunction with ferroptosis?
- Will cancer cells have different ferroptosis tendency if they have different energy resource, either relying more on mitochondrial respiration or glycolysis?

## Introduction

**Fig. 1:**
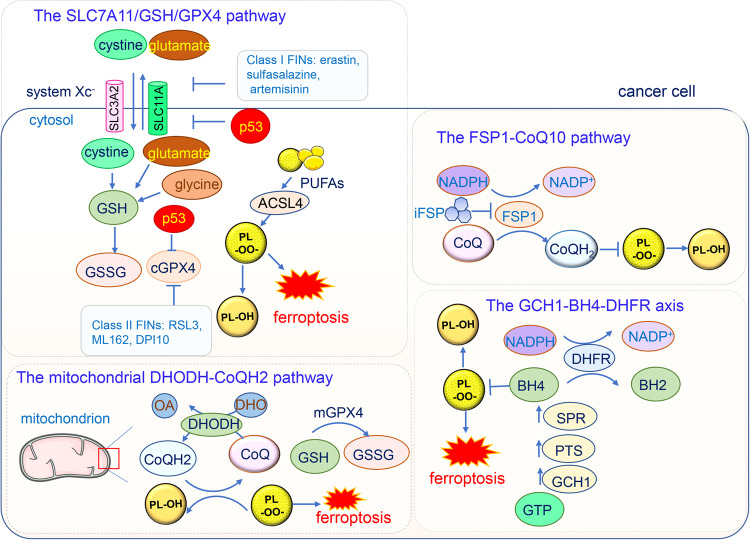
The major signaling pathways in ferroptosis. The GPX4-GSH pathway occurs mainly in the cytoplasm (cGPX4) and mitochondria (mGPX4). This axis can be inhibited by FINs and tumor suppressor p53. The FSP1-CoQ pathway occurs in the plasma membrane which can be inhibited by iFSP. The GCH1-BH4 pathway starts from the substrate GTP and inhibits lipid peroxidation by BH4–BH2 cycle. The DHODH-CoQH2 pathway is located at the outer surface of inner mitochondrial membrane, which catalyzes the conversion of DHO to OA while simultaneously refreshing CoQH2 to clear lipid radicals. SLC7A11 solute carrier family 7 member 11, cGPX4 cytoplasmic glutathione peroxidase 4, GSH glutathione, GSSG glutathione disulfide, PL-OO· Phospholipid hydroperoxide, FIN ferroptosis inducing, PULA long-chain polyunsaturated fatty acids, ACSL4: acyl-CoA synthetase long-chain family member 4, FSP1: ferroptosis suppressor protein 1; iFSP1 FSP1 small-molecule inhibitor, GCH1 cyclohydrolase-1, GTP cyclohydrolase-1, PTS 6-pyruvoyltetrahydropterin synthase, SPR sepiapterin reductase, DHFR dihydrofolate reductase, DHODH dihydroorotate dehydrogenase, OA orotate, DHO dihydroorotate, mGPX4 mitochondrial glutathione peroxidase 4.

Regulated cell death, mainly including apoptosis, entosis, necroptosis, and pyroptosis, plays a critical role in maintaining homeostasis under different disregulating conditions in the intracellular or extracellular microenvironment [1]. Ferroptosis was discovered by Dixon et al. in 2012, who found that the oncogenic RAS-selective lethal small-molecule erastin triggered a unique iron-dependent form of nonapoptotic cell death that they termed as ferroptosis [2]. Since its discovery, ferroptosis has been extensively studied. Ferroptosis is characterized by excessive intracellular iron ions and accumulated lethal lipid-based reactive oxygen species (ROS). The biochemical process underlying ferroptosis mainly involves intracellular glutathione (GSH) depletion and decreased glutathione peroxidase 4 (GPX4) activity. Excessive lipid peroxides cannot all be metabolized by the GPX4-catalyzed reduction reaction, leading to the accumulation of ROS [3]. The small-molecule erastin triggers ferroptosis by inhibiting the activity of the cystine-glutamate antiporter (SLC7A11, system Xc-), inducing the depletion of cellular cysteine and GSH and leading to the collapse of cellular redox homeostasis [4, 5]. Besides iron and ROS, ferroptosis is regulated by other pathways such as the p53 pathway in cancer, it can be suppressed by several pathways including ferroptosis suppressor protein 1 (FSP1)-CoQ10 pathway [6, 7], GCH1-BH4 pathway [8], and the DHODH-CoQH2 system [9, 10] (Fig. 1).

Mitochondria are not only the main energy resources for cells, but also signaling organelles involved in physiological and pathological processes [11–14]. Paradoxically, mitochondria play a central role in apoptotic cell death [15], pyroptosis [16], necroptosis [17], ferroptosis [4], and other forms of cell death. The classical mitochondrial pathway in apoptosis is related to the increased permeability of mitochondrial outer membrane, which enables some soluble proteins such as cytochrome *c* in the mitochondrial intermembrane space to be released into the cytoplasm, then apoptotic signaling pathways are activated, causing cell death. In pyroptosis, mitochondrial outer membrane permeabilization (MOMP) participates in the leakage of cytochrome c from the mitochondrion to the cytosol and subsequently activates caspase proteases and causes cell death [18]. In ferroptosis, mitochondria experience a morphological change including increased membrane density and reduced or vanishing mitochondrial cristae [4]. Moreover, mitochondria energy metabolism changes in ferroptosis, the oxidative phosphorylation synthesis and ATP production are increased correspondingly the glycolysis is decreased [2, 19]. Furthermore, the oxidative stress level is increased, and excessive oxidative stress induces irreversible damage to mitochondria, diminishing organelle integrity, ultimately leading to energy depletion and cell death. In addition to morphology and energy metabolism changes, ROS production, the mitochondrial membrane potential, mitochondrial fusion and fission, and mitophagy are involved in ferroptosis. In addition, some ferroptosis inhibitors target mitochondria. Ferroptosis is closely associated with cancer progression and metastasis, Mesenchymal cancer cells which are metastasis-prone cells are often resistant to various treatments but are highly sensitive to ferroptosis [20–22]. However, cancer cells dying from ferroptosis impede dendritic cell-mediated anti-tumor immunity [23]. Inducing or prohibiting ferroptosis in tumor cells shows vastly promising potential in treating drug-resistant cancers. Various ferroptosis inducers and inhibitors such as ferrostatin-1 have been developed and ferroptosis has recently been recognized as a novel cancer-elimination target. The in-depth comprehension of ferroptosis and its intercellular consequences may lead to the identification of novel therapeutic targets for cancers. The diverse roles played by mitochondria will be elucidated to exhibit the crosstalk between mitochondria and ferroptotic pathway components, which helps in understanding the importance of mitochondria in ferroptosis in cancer cells.

## Regulation of ferroptosis

Ferroptosis is regulated by many metabolic pathways. We will discuss the classical and newly discovered pathways suppressing ferroptosis. Ferroptosis monitoring mechanisms mainly can be divided into three types: (1) one is mediated by GPX4, which prevents ferroptosis by reducing phospholipid peroxide to corresponding phospholipid alcohol; (2) one is mediated by FSP1, DHODH, NOS2, GCH1 and other enzymes. These enzymes produce metabolites with free radical capture antioxidant (RTA) activity, thus terminating phospholipid peroxidation and inhibiting ferroptosis; (3) and the new discoveries that sex hormones suppress ferroptosis mediated by MBOA1/2.

### The GSH/GPX4 pathway suppresses ferroptosis

GPX4 is a selenium-containing antioxidative enzyme, that belongs to a family of eight phylogenetically related glutathione peroxidases(GPX1-8) which participate in the cellular antioxidative defense system [24]. In contrast to other GPX family proteins which generally reduce H_2_O_2_ and small hydroperoxides, GPX4 reduces the hydroperoxides in complex lipids, such as hydroperoxides in phosphatidylcholine, cholesterols, and cholesteryl ester, even when those are integrated into membranes or lipoproteins [25]. Notably, GPX4 is the rate-limiting enzyme critical for mammalian cell survival, and its selenium binding site is indispensable for cell resistance to ferroptosis [26].

There are three distinct isoforms of GPX4, cytosolic (cGPX4), mitochondrial (mGPX4), and sperm nuclear GPX4 (snGPX4), which are generated via its splicing variants. Unless specified otherwise, in this review, we mainly discuss cGPX4 due to its ubiquitous activity in ferroptosis. Homozygous ablation of the GPX4 gene in mice led to high levels of oxygen radicals and early embryonic lethality [27]. Even in conditional GPX4 knockout mouse models, excessive lipid peroxidation and oxidative stresses were detrimental to tissues [28–31]. In contrast, overexpression of GPX4 inhibited cell death induced by multiple stimuli [32].

GPX4 leverages the activity of a wide range of reductants to detoxify the oxidative radicals and is thus the main inhibitor of ferroptosis [3, 33, 34]. GSH is the major reducing substrate for GPX4. The tripeptide GSH is derived from cysteine, glutamate, and glycine, among which intracellular cysteine is the rate-limiting precursor and is transported by System Xc^−^, a membrane cysteine-glutamate antiporter. Thus, the System Xc^−^, GSH, and GPX4 (together forming the Xc^−^/GSH/GPX4 axis) constitute the core ferroptosis regulating system [35]. In addition to GSH, GPX4 leverages other substrates such as cysteine, thiols, and certain proteins in the body, enhancing its high anti-ferroptotic effectiveness [24]. Ferroptosis-inducing (FIN) compounds are categorized into two classes according to their mechanisms of action on GPX4 activity. Class I FINs, including erastin, sulfasalazine, and artemisinin (as well as its derivatives), inhibit cystine uptake via System Xc^−^ [2, 36] which indirectly decreases GPX4 activity by depleting GSH. Class II FINs such as RAS-selective lethal 3 (RSL3) [37], ML162 [38], and DPI10 [39], covalently bind to GPX4 and decrease its enzymatic activity [33]. These GPX4 inhibitors result in elevated ROS levels and trigger cell ferroptosis.

Importantly, GPX4, as a central regulator of ferroptosis, connects other cellular molecules and pathways with ferroptosis. Acyl-CoA synthetase long-chain family member 4 (ACSL4) is involved in cell ferroptosis sensitivity, and directly incorporates long-chain polyunsaturated fatty acids (PUFAs) into phospholipid membranes, which trigger the lipid peroxidation chain reactions that induce ferroptosis [40]. The metabolite isopentenyl pyrophosphate (IPP) promotes selenocysteine tRNA maturation and GPX synthesis, thereby preventing ferroptosis initiation [26]. The upregulation of Sirtuin 3 (SIRT3), which is an NAD^+^-dependent mitochondrial protein deacetylase, induces ferroptosis in trophoblastic cells through GPX4 inhibition [41]. Serum autoantibodies and interferon-α suppressed GPX4 expression by promoting the binding of its promoter and suppressor cAMP response element modulator a (CREMa), and eventually drove neutrophil ferroptosis in the lupus-prone mice [42]. Nuclear factor erythroid 2-related factor 2 (Nrf2), a stress-inducible transcription factor, protects cells from ferroptosis by directly or indirectly regulating GPX4 protein content. Nrf2 controls the expression of GPX and a number of glutathione synthesis- and metabolism-related enzymes [43]. Overall, GPX4 is crucial for ferroptosis and cannot be replaced by any other redox-active enzymes in the ferroptotic pathway.

### The FSP1-CoQ10 pathway suppresses ferroptosis

Although we cannot sufficiently emphasize the importance of GPX4, other GPX4-independent pathways are involved in the regulation of ferroptosis. Ferroptosis suppressor protein 1 (FSP1, also known as flavoprotein apoptosis-inducing factor mitochondria-associated 2, AIFM2) protects cells against ferroptosis regardless of the cell GSH level, GPX4 activity level, or p53 status, and its activity is mediated by extramitochondrial ubiquinone (also known as coenzyme Q10, CoQ10) [6]. The reduced form of CoQ10, ubiquinol (CoQH_2_), prevents lipid peroxidation, and FSP1 maintains the regeneration of CoQ10 in an NADPH-dependent manner. The canonical N terminal myristoylation motif of FSP1 is required for FSP1 recruitment to the plasma membrane, where it confers ferroptosis resistance [7].

Disruption of the FSP1-CoQ10 pathway induces ferroptosis. As induced by either selective small-molecule inhibitor iFSP1 or genetic deletion, FSP1 loss-of-function promotes ferroptosis. Interestingly, FSP1 inhibition selectively sensitizes cells to FINs but not other cytotoxic compounds such as cisplatin [6]. Another ferroptosis inducer FIN56, which induces GPX4 protein degradation, also interferes with the FSP1-CoQ10 pathway activation by activating squalene synthase in the mevalonate pathway and thus suppressing CoQ10 activity [44]. Squalene synthase inhibitors alleviate the lethality induced by FIN56 because they increase the levels of mevalonate metabolites such as farnesyl pyrophosphate (FPP) and CoQ10 [44]. FSP1 overexpression failed to protect CoQ10-deprived COQ2-KO cells from ferroptosis, in this experiment, 4-hydroxybenzoate polyprenyltransferase (COQ2) was depleted, which abrogated the first step in CoQ10 biosynthesis [6].

### The GCH1-BH4-DHFR axis inhibits ferroptosis

The guanosine 5′-triphosphate (GTP) cyclohydrolase-1 (GCH1) tetrahydrobiopterin (BH4) pathway is a crucial GPX4-independent ferroptosis regulation system. The biosynthesis of BH4 from its precursor GTP requires three enzymatic steps catalyzed by GCH1, 6-pyruboyltetrahydropterin synthase (PTS) and sepiapterin reductase (SPR), among which the GCH1 is the rate-limiting enzyme [45]. GCH1 expression levels largely determine the degree of the cell resistance to ferroptosis. Genetic or pharmacological inhibition of GCH1 results in BH4 insufficiency which drives cell toward peroxidation accumulation and ferroptosis [46]. In contrast, overexpression of GCH1 selectively enhances the BH4 biosynthesis and decreases ROS production [47, 48].

BH4, paired with dihydrobiopterin (BH2), forms a redox cycle to diminish endogenous oxidative radicals and protect lipid membranes to inhibit ferroptosis [49], these effects are observed when BH4–BH2 is administered alone and show synergic effects when administered with vitamin E (also known as a-tocopherol). The regeneration of BH4 from BH2 is controlled by dihydrofolate reductase (DHFR) in which NADP^+^/NADPH is the cofactor. Supplementation with BH4, but not BH2, directly reversed the DHFR inhibition-induced ferroptosis in GCH1-knockout cells [46, 49]. In addition, BH4 may promote the production of CoQ10 via its effect on the synthesis of its precursor 4-OH-benzoate. Taken together, these mechanisms link the GCH1-BH4-DHFR axis with the FSP1-CoQ10 axis, which coordinatively and precisely control ferroptosis.

### The mitochondrial DHODH-CoQH_2_ system inhibits ferroptosis

In contrast to the aforementioned pathways, the dihydroorotate dehydrogenase (DHODH)-CoQH2 regulatory system blocks the mitochondrial lipid peroxidation and thus ferroptosis [50]. DHODH is located at the outer surface of the inner mitochondrial membrane (IMM), where it catalyzes the ubiquinone-mediated oxidation of dihydroorotate (DHO) to orotate (OA) and simultaneously inhibits ferroptosis [50]. DHODH-CoQH_2_ and mGPX4 function individually but both reduce CoQ to CoQH_2_ [51]. Supplementation with DHO and OA attenuates and sensitizes, respectively, the GPX4 inhibition-induced increase in the anti-ferroptosis defense network in mitochondria [52].

### Sex hormones inhibit ferroptosis of cancer cells via MBOAT1/2 mediated by phospholipid remodeling

In May.2023, Jiang lab found Membrane-bound O-acyltransferases1/2 (MBOAT1/2) are the ferroptosis suppressors by a whole-genome CRISPR activation screen [53]. MBOAT1 and MBOAT2 inhibit ferroptosis by remodeling phospholipids, which is a new ferroptosis monitoring mechanism independent of GPX4 or FSP1. They are upregulated by estrogen receptor (ER) and androgen receptor (AR) respectively. ER or AR antagonists combined with ferroptosis induction inhibit the ER^+^ cancer or AR^+^ prostate tumor growth [53].

### Tumor suppressors regulate ferroptosis

Tumor suppressors such as NF2 suppress ferroptosis, whereas other suppressors such as MLL4 FBW7, and BAP1 promote ferroptosis [54]. MLL4 is an epigenetic regulator that is often rendered dysfunctional in cutaneous squamous cell carcinomas [55]. MLL4 deficiency triggers the upregulation of SLC7A11 and GPX4, which drive resistance to ferroptosis and loss of the lipoxygenases such as arachidonate 12-lipoxygenase (ALOX12), ALOX12B and ALOXE3 [55]. Moreover, genetic inactivation of the tumor suppressor NF2, a frequent tumorigenic event in mesothelioma, renders cancer cells more sensitive to ferroptosis, malignant mutations in NF2–YAP signaling can be used to predict the responsiveness of cancer cells to future ferroptosis-inducing therapy [56]. FBW7 has been shown to regulate lipid peroxidation and enhance ferroptosis in pancreatic cancer and to inhibit stearoyal-CoA desaturase (SCD1) activity [57]. In addition, the tumor suppressor BAP1 decreases H2Aub occupancy on the SLC7A11 promoter and represses SLC7A11 expression in a deubiquitinating-dependent manner, that is, BAP1 inhibits cystine uptake by repressing SLC7A11 expression, leading to elevated lipid peroxidation and ferroptosis rates [54].

In recent years, the role of the tumor suppressor gene p53 in ferroptosis has become a topic of great interest. p53 mutations have been tightly linked with tumorigenesis [58]. Diverse stress signals produced during tumor initiation, such as those emitted by telomere attrition, DNA damage, nutrient deprivation, hypoxia, oxidative stress, and hyperproliferation, *etc*, can activate p53 and initiate the p53-dependent tumor suppression responses such as DNA repair, cell-cycle inhibition, senescence, autophagy, apoptosis [59]. The inactivation/mutations of the p53 gene are detrimental and connected with the majority of human cancers [60]. The role of the classical tumor suppressor p53 on ferroptosis is complicated as it either augments or suppresses ferroptosis depending on the conditions.

The Gu group first revealed that p53 promotes ferroptosis via a novel tumor suppression mechanism that is independent of canonical p53 functions in cell-cycle arrest and senescence [61, 62]. P53 activation can sensitize cells to ferroptosis after ROS-induced stress by repressing the expression of GPX4 and system Xc^−^ component SLC7A11, which limits GPX4 substrate availability [61, 63–66]. SLC7A11 is highly expressed in human tumors, and its overexpression inhibited ROS-induced ferroptosis and abrogates p53-mediated tumor growth suppression in xenograft models [61]. Knockdown of p53 expression can reverse the suppression of SLC7A11 and GPX4 expression [67]. P53^3KR^ mutant (Arginine R117/R161/R162) retains the ability to promote ferroptosis while failing to induce cellular apoptosis [61]. Moreover, the p53^4KR98^ mutant (R98/R117/R161/R162) failed to repress SLC7A11 transcription or induce ferroptosis [68], suggesting a critical role of p53 acetylation in SLC7A11-related ferroptosis. P53 not only suppresses SLC7A11 activity but also activates ALOX12 [69]. The ALOX12 is essential for p53-dependent but not erastin-induced ferroptosis as p53 releases the lipoxygenase ALOX12 from the inactive ALOX12-SLC7A11 complex [69]. In addition, p53-mediated ferroptosis can be elicited in an SLC7A11/GPX4-independent manner. The spermidine/spermine N1-acetyltransferase 1 (SAT1) is highly induced by p53 and loss of SAT1 expression partially abrogates p53-mediated ferroptosis. SAT1-induced ferroptosis relies on the expression of ALOX15 but not the regulation of SLC7A11 and GPX4, even though ALOX15 belongs to the same mammalian lipoxygenase family as ALOX12 [70]. The calcium-independent phospholipase iPLA2b is sufficient to detoxify the peroxidized lipids to suppress p53-driven ferroptosis upon ROS-induced stress [71]. Compared with ferroptosis dependence on GPX4, neither cell homeostasis nor embryonic development depends on iPLA2b, but it is critical for ferroptosis induction during stress responses [71].

Conversely, p53 can suppress ferroptosis through the following mechanisms, including but not limited to the direct inhibition of dipeptidyl peptidase 4 (DPP4) and the induction of cyclin-dependent kinase inhibitor 1A (CDKN1A/p21) expression [72]. DPP4 has peptidase activity but this is not essential for ferroptosis induction. The loss of p53 enhances erastin-induced ferroptosis as DPP4 accumulation in the cell membrane triggers lipid peroxidation [64]. In addition, the expression of CDKN1A, a key transcriptional target of p53 in terms of cell-cycle arrest, delays the onset of ferroptosis through system Xc^−^ regulation or slower cystine deprivation [73]. Taken together, the evidence suggests that the regulation of ferroptosis by p53 is multifactorial and bidirectional, and a better understanding of p53-related ferroptosis may lead to new treatments for human diseases, especially those related to p53 mutations.

The research on the ferroptosis inhibition are being explored. Besides the above pathways, more proteins or pathways are discovered to suppress ferroptosis. For example, it is found that G3P dehydrogenase 2 (GPD2) worked together with GPX4 to suppress ferroptosis [74]. Nedd4 suppresses ferroptosis by ubiquitylating VDAC 2/3 in melanoma [75].

## Role of mitochondria in ferroptosis

Mitochondria play critical roles in metabolic plasticity in malignant cells, as well as in the regulation of many types of RCDs including ferroptosis. The role of mitochondria in ferroptosis has been explored in 2019 by Gao. et al. [4]. Mitochondria either prompt or suppress ferroptosis. The high level of ROS, the starvation due to decomposition of mitochondrial glutamine, and the high concentration of iron ions induce ferroptosis. Conversely, the intact and normal mitochondrial function resist ferroptosis, both β-oxidation and Fe/S cluster formation augment the resistance of cells to ferroptosis. We mainly summarize the diverse roles of mitochondria by presenting new discoveries made in the past three years.

### Mitochondrial iron metabolism regulates ferroptosis

Mitochondria, as diversified organelles, perform numerous bioenergetic, biosynthetic, and regulatory functions and play a central role in iron metabolism. Extracellular iron is taken up by cells and transported to the mitochondria, where it is utilized for the synthesis of cofactors including heme and iron-sulfur (Fe/S) clusters essential to the function of enzymes involved in oxidation-reduction reactions, DNA synthesis and repair, and a variety of other cellular processes [76]. Fe/S cluster-containing proteins, such as NADH (ubiquinone oxidoreductase) and heme-containing proteins such as cytochrome c, cytochrome c oxidase, and succinate dehydrogenase, are components of the IMM complexes of the ETC, the iron balance is essential for the ATP production function [77]. The influx of cytoplasmic iron into mitochondria and the utilization of the free iron were mediated by mitoferrin, endosomes, or other mechanisms [78]. Iron is the most prevalent metal inside the mitochondria [79].

Mitochondrial iron is metabolized mainly in the mitochondrial matrix; therefore, iron in the cytosol needs to be transported to the mitochondrial matrix from the outer membrane (OMM). Voltage-dependent anion channels (VDACs) are important outer membrane proteins in mitochondria that are responsible for controlling the exchange of ions and metabolites between the cytosol and mitochondria [80, 81]. They regulate the influx of iron in mitochondria. After erastin treatment, VDAC 2/3 channels open and iron accumulates in mitochondria leading to iron-dependent ferroptosis [82]. Iron is transported across the inner membrane by transporter mitoferrin 1(Mfrn1) and its homolog mitoferrin 2 (Mfrn2). Dysfunctional iron transport results in iron accumulation and oxidative damage [83]. Iron accumulation leads to ferroptosis and is evident in different diseases [84]. Moreover, the iron-sulfur protein (2Fe-2S) NEET mediates the export of sulfur ions and iron between the cytosol and the mitochondria. Depletion of mitoNEET leads to mitochondrial iron accumulation and the generation of mitochondrial lipid peroxides contributing to ferroptosis [85, 86]. In addition, the mitochondrial Ca^2+^ uniporter (MCU) and mitochondrial permeability transition pore (mPTP) have been proposed as pathways for iron entry into mitochondria [87, 88], and blocking MCU by Ru360 reduced mitochondrial iron uptake and showed higher efficacy than blocking the mPTP with cyclosporin A as it leads to more profound decreases mitochondrial ROS levels and increased mitochondrial function, indicating that MCU may be a crucial portal for iron uptake by mitochondria [89]. A similar result was achieved by adding iron chelator deferoxamine to cell cultures [87, 90].

**Fig. 2:**
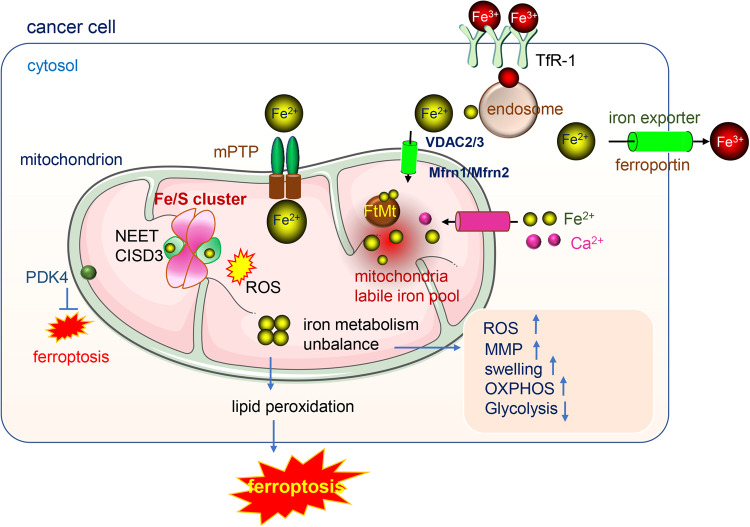
The iron metabolism in mitochondria. Fe^3+^ enters cytosol via the transporter TfR1, with the help of endosome and other transporters, the Fe^2+^ generated, Fe^2+^ enters into mitochondria mainly via three passways: (1) by VDAC 2/3 and Mfrn1/Mfrn2 (2) by the mPTP (3) by the Ca^2+^ uniporter. The mitochondrial iron metabolism mainly occurs in the mitochondria matrix. The iron in mitochondria is utilized for the synthesis of cofactors including heme and iron-sulfur (Fe/S) clusters essential to the function of enzymes involved in oxidation-reduction reactions. Mitochondria contain a labile iron pool and the balance of iron homeostasis is controlled by FtMt. The dysfunction of mitoNEET or unbalance of iron metabolism lead to the accumulation of iron in mitochondria, the increase of ROS, MMP and mitochondria swelling and the energy production change.

Mitochondria maintain a labile iron pool and iron homeostasis is controlled by FtMt [91]. FtMt functions as ferroxidase and it shows an iron-binding affinity similar to that of ferritin in the cytosol [92]. Mitochondrial ferritin protects against mitochondrial ROS accumulation [93]. Dysfunction of FtMt leads to the accumulation of mitochondrial free iron and ROS and ferroptosis [94]. Iron overload leads to mitochondrial dysfunction as indicated by decreased mitochondrial respiration, increased mitochondrial ROS levels, mitochondrial membrane potential depolarization, and mitochondrial swelling [90, 95]. Hence, when iron hemostasis is interrupted, excessive iron may injure proteins, lipids and DNA within mitochondria and reduce ATP production, leading to energy stress and ferroptosis (Fig. 2).

### The mitochondrial OXPHOS pathway participates in ferroptosis

#### Cells leverage different energy resources during ferroptosis

Metabolic plasticity is the essential ability of cells to adapt to the environment. The metabolic switch from mitochondrial respiration to aerobic glycolysis provides flexibility to sustain cellular energy. Recent reports have revealed that cellular energy metabolic pathways such as glycolysis, the pentose phosphate pathway (PPP), and the tricarboxylic acid (TCA) cycle are involved in the regulation of key ferroptosis marker levels. For example, they reduced nicotinamide adenine dinucleotide phosphate (NADPH), GSH, and ROS levels, thereby playing potential regulatory roles in ferroptosis [96]. During ferroptosis, when cancer cells are under ROS-induced stress and the antioxidant defense is dysfunctional in the tumor cellular environment, cancer cells shift energy from the OXPHOS pathway to the glycolysis pathway to support vital biological events such as macromolecular biosynthesis, membrane-protein integration and to maintain cross-membrane ion gradients and DNA replication [96]. In neuronal cells, the activation of small-conductance calcium-activated K^+^ (SK) channels modulates mitochondrial respiration and protects neuronal cells from oxidative death, inducing a shift to glycolysis and increasing the resilience of neuronal cells against ferroptosis induced by erastin [97].

#### Energy stress inhibits ferroptosis via AMPK pathway action

**Fig. 3:**
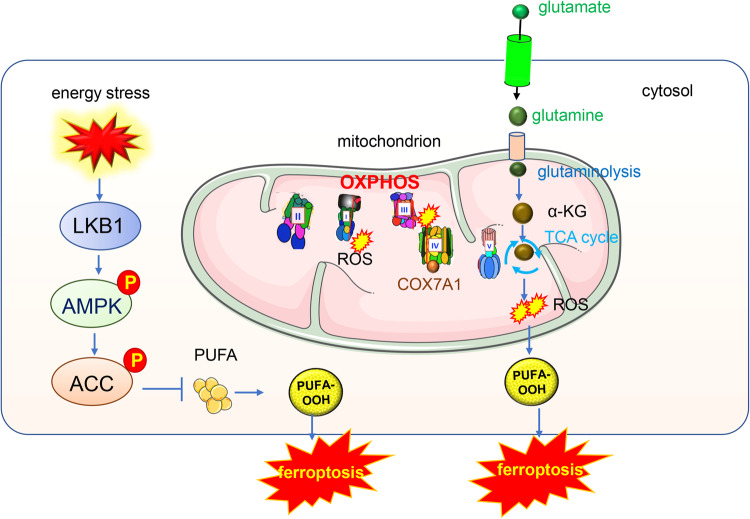
Mitochondria OXPHOS pathway and TCA cycle participate in the ferroptosis. AMPK is the energy sensor, cancer cells with high basal AMPK activation are resistant to ferroptosis and AMPK inactivation sensitizes these cells to ferroptosis. Mechanistically, AMPK regulates ferroptosis through acetyl-CoA carboxylase (ACC) and polyunsaturated fatty acid (PUFA) biosynthesis. TCA metabolites such as α-KG and its downstream products succinic acid and fumaric acid could all enhance the ferroptosis induced by cysteine depletion.

Cancer cells acquire energy through oxidative phosphorylation (OXPHOS) or glycolysis. The OXPHOS pathway functions in mitochondria and is the main ATP-producing system. Energy stress, which is characterized by the depletion of intracellular ATP and a corresponding increase in intracellular AMP levels inhibits ferroptosis. AMP-activated protein kinase (AMPK) is an energy sensor [98], cancer cells with high basal AMPK activation are resistant to ferroptosis, while AMPK inactivation sensitizes these cells to ferroptosis [99]. Mechanistically, the LKB1-AMPK axis negatively regulated ferroptosis by inhibiting fatty acid synthesis, and the loss of function of tumor suppressor LKB1 enhanced human non-small cell lung cancer cells’ sensitivity to ferroptosis [100]. AMPK regulates ferroptosis through acetyl-CoA carboxylase (ACC) and polyunsaturated fatty acid (PUFA) biosynthesis [101]. The LKB1-AMPK pathway negatively regulates ferroptosis by inhibiting the phosphorylation of ACC1 [100] (Fig. 3).

#### The electron transport chain (ETC) regulates ferroptosis

Mitochondrial ETC activity is not only essential for ATP production but is also required for the generation of sufficient lipid ROS to initiate ferroptosis. Most ROS are generated by the ETC during electron transport. When any of the key ETC genes are deficient or ETC action is inhibited, the number of ROS increase, and high levels of accumulated ROS may induce ferroptosis. In addition, enhanced ETC activity also affects cancer cell sensitivity to ferroptosis; for example, COX7A1, a subunit of cytochrome c oxidase, suppressed mitochondrial dynamics as well as mitochondrial biogenesis and mitophagy by blocking autophagic flux, which strengthened the sensitivity of NSCLC cells to cysteine deprivation-induced ferroptosis [102]. Moreover, pannexin 1 (PANX1), an ATP-releasing pathway family protein, showed proapoptotic effects in kidney injury cells. PANX1 deletion protects against renal ischemia/reperfusion injury (IRI) by regulating ferroptotic cell death [103].

The mitochondrial NEET protein family plays a key role in mitochondrial labile iron stores and ROS homeostasis. NEET proteins carry labile [2Fe–2S] clusters, which can be transferred to apo-acceptor proteins [104]. CISD3, a member of the NEET protein family, is generally elevated in various human cancers which are consequently associated with a poor prognosis. CISD3 depletion leads to a metabolic reprogramming toward glutaminolysis required for the fuel of mitochondrial OXPHOS. Knocking down the CISD3 significantly accelerates lipid peroxidation and free iron accumulation initially triggered by Xc^−^ inhibition or cystine deprivation, causing ferroptotic cell death [105].

### The mitochondrial TCA cycle participates in ferroptosis

Mitochondria link the TCA cycle and fatty acid oxidation (FAO). Glutaminolysis and TCA cycle are involved in ferroptosis, but the underlying metabolic process remains unclear. Various TCA metabolites and enzymes have close relations with ferroptosis, the expression level of these key enzymes determines the sensitivity of cancer cells to ferroptosis.

Glutamine is required for ferroptosis and glutamine synthase 2 (GLS2) is a key regulator of glutaminolysis and has been shown to promote ferroptosis [106]. GLS2 has been previously implicated in activities consistent with tumor suppression. The degradation of glutamine required GLS, glutamate dehydrogenase (GDH) and GOT2 to provide fuel for TCA cycle and lipid biosynthesis. TCA cycle metabolites such as α-ketoglutarate (α-KG) mimic the function of glutamine in ferroptosis. α-KG and its downstream products such as succinic acid and fumaric acid, all enhance ferroptosis induced by cysteine depletion [4] (Fig. 3). Citrate synthase regulates the synthesis of fatty acids, and the acyl-CoA syntheses family member 2(ACSF2) modulates the activation of fatty acid. These fatty acid products function as specific lipid precursors in FAO [4]. Moreover, the pyruvate dehydrogenase kinase 4 (PDK4), located in the inner membrane of mitochondria, mediates metabolic resistance to ferroptosis by suppressing pyruvate oxidation and fatty acids synthesis, which facilitates lipid peroxidation-dependent ferroptotic death [107].

Malic enzyme 1(ME1) is frequently overexpressed in cancers, and catalyzes the reversible oxidative decarboxylation of malate to pyruvate, yielding NADPH from NADP^+^, ME1 as a major cytoplasmic source of NADPH. In Synovial Sarcoma, the absence of ME1 shifts antioxidant system dependence and increases sensitivity to ferroptosis induced by ACXT-3102 [108].

Fumarate hydratase (FH) is a TCA cycle enzyme. Hereditary leiomyomatosis and renal cell cancer (HLRCC) are hereditary cancer syndromes characterized by the inactivation of FH. The inactivation of FH is induced and leads to cell death via ferroptosis. Mechanistically, the FH^−/−^ sensitivity to ferroptosis has been attributed to dysfunctional GPX4, the primary cellular defender against ferroptosis [109].

Isocitrate dehydrogenase (IDH1 and IDH2) mutations are evident in many types of cancers including glioblastoma [110], and acute myeloid blood (AML) [111]. IDH1 mutation in cholangiocarcinoma inhibits tumor progression by sensitizing cells to ferroptosis [112]. Mitochondrial NADP^+^-dependent IDH2 is a major enzyme that produces NADPH, which is a crucial driver of mitochondrial GSH turnover, and downregulation of IDH2 sensitizes cancer cells to erastin-induced ferroptosis [113]. Tumor-derived IDH1 mutation sensitizes cells to ferroptosis, and inhibition of mutant IDH1 produces the oncometabolite D-2-hydroxyglutarate (D-2-HG), which confers resistance to erastin-induced ferroptosis. Mechanistically, mutant IDH1 reduces the protein level of GPX4 and promotes the depletion of glutathione, thus inducing ferroptosis [114].

Lactate regulates ferroptosis in liver cancer cells. Notably, lactate-enriched hepatocellular carcinoma (HCC) cells exhibit enhanced resistance to ferroptotic damage. Moreover, monocarboxylate transporter 1 (MCT1)-mediated lactate uptake promotes ATP production and deactivates AMPK, which increases the expression of sterol regulatory element-binding protein 1 (SREBP1) and the downstream stearoyl-coenzyme A (CoA) desaturase-1 (SCD1) to enhance the production of anti-ferroptosis monounsaturated fatty acids [115].

### Bursts in Ros in mitochondria induce ferroptosis

ROS are the natural byproducts of numerous enzymatic reactions in cells. They are generated by enzymes such as nicotinamide adenine dinucleotide (NADPH) oxidases. ROS production modulates cellular homeostasis and ROS signaling participates in normal physiological processes and contributes to metabolic dysfunction [116]. In the mitochondrial ETC, Complex I and Complex III are the main sites of ROS generation. Among typical ROS, the superoxide anion (O_2_^−^) is produced from reverse electron transfer through Complex I (NADH) after O_2_ interacts with reduced flavin mononucleotide (FMN); when the matrix NADH/NAD^+^ ratio is high, the O_2_^−^ generated is released into the matrix [117]. Mitochondrial complex II can generate ROS when the functions of complex I and III are inhibited, and ROS may be produced via the electron transfer from succinate or in the reverse direction when electrons are provided from a reduced ubiquinone pool [118].

The predominant ROS-scavenging system includes the glutaredoxin, glutathione and thioredoxin systems [119]. H_2_O_2_ is decomposed into oxygen and water via the GSH redox system which includes glutathione reductases, peroxidases GPX (Glutathione peroxidase) and peroxiredoxins [120]. A dysfunctional ROS-scavenging system leads to excessive ROS accumulation in cells and lipid peroxidation, mainly in the cell membrane resulting in a loss of membrane properties and ferroptosis. ROS accumulation is considered as a hallmark of ferroptosis.

Excessive ROS damage lipids, nucleic acids and proteins, leading to DNA damage and protein dysfunction, increasing the rates of tumor initiation and progression. Oxidative stress is a result of an imbalance between ROS levels and antioxidative defense systems [121]. Oxidative stress drives the expression of cancer-suppressing genes. ROS suppress p53 expression and promote cancer progression [122]. An increase in ROS and a reduction in GSH have been identified in the development of head-and-neck carcinoma [123]. In addition to cancers, ROS regulate ferroptosis in other diseases. For example, in Alzheimer’s disease, NADPH oxidase 4 (NOX4) is a major enzyme in ROS production, and the overexpression of NOX4 significantly increased the impairment of mitochondrial metabolism by inhibiting mitochondrial respiration and ATP production via its effect on ETC complexes in human astrocytes. In this case, NOX4 promoted the ferroptosis of astrocytes via oxidative stress-induced lipid peroxidation induced after mitochondrial metabolism was dysregulated [124].

The accumulation of ROS in mitochondria triggers ferroptosis, and this process is inhibited by antioxidants that target mitochondria. In erastin- or doxorubicin-induced ferroptosis, lipid ROS accumulate, which partially explains the consumption of GSH in mitochondria during ferroptosis. Hence, ROS scavengers such as MitoTEMPO and mitoquidone are used to inhibit ferroptosis in various cancer cells. In addition, microsomal glutathione-S-transferase 1 (MGST1) is a major antioxidant located in mitochondria and the ER, and it limits lipid peroxidation and ferroptosis by binding to ALOX5. MGST1 is considered a novel therapeutic target in pancreatic cancer due to the role it plays in the ferroptosis of pancreatic cancer cells [125].

### Mitochondrial membrane potential regulates ferroptosis

The mitochondrial membrane potential (MMP, ΔΨm) generated by proton pumps (Complexes I, III and IV) is essential to energy storage after oxidative phosphorylation. During mitochondrion-mediated apoptosis, the MMP decreases before the eventual cell death. However, in ferroptosis induced by erastin or cystine starvation, cells exhibit increased mitochondrial membrane potential (hyperpolarization), greater membrane density and a corresponding reduction in membrane volume [4]. Treatment with the mitochondrial uncoupler CCCP disrupts the MMP and completely blocks lipid ROS accumulation and ferroptosis. Disrupting MMP by CCCP rescues cells from ferroptotic cell death without compromising long-term cell viability. The MMP hyperpolarization of ferroptosis reflects an increase in mitochondrial ETC activity and subsequent lipid ROS generation and accumulation, and excessive ROS leads to exacerbated ferroptosis, which explains the role of MMP in ferroptosis.

### Mitochondrial dynamics during ferroptosis

Mitochondria are highly dynamic organelles. Mitochondria undergo fusion and fission to maintain their integrity and homeostasis. Mitochondrial fusion refers to the outer membrane and inner membrane in two mitochondria joining together via the action of the three important mitofusin proteins: mitofusin 1 (MFN1), mitofusin 2 (MFN2) and optic atrophy protein 1 (OPA1). Mitochondrial fission is regulated by dynamin-related protein 1 (Drp-1) [126, 127]. Mitochondria fusion and fission have close relation with iron metabolism and ferroptosis. Iron overload disrupts mitochondrial dynamics, interfering with the balance between mitochondrial fission and fusion [95]. Genetic depletion of MFN1/2 reduced the sensitivity of pancreatic cancer cells to ferroptosis [128].

**Fig. 4:**
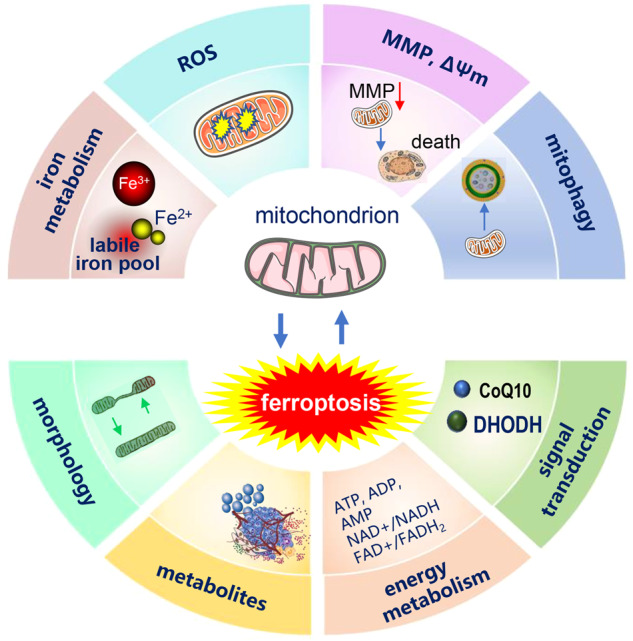
Crosstalk between mitochondria and ferroptosis. The mitochondria iron metabolism and ROS production affect ferroptosis, the overloading of iron and the excessive ROS induces ferroptosis. In the process of ferroptosis, the mitochondria morphology changes. Critical enzymes working in mitochondria such as DHODH and CoQ10 inhibit ferroptosis.

The interaction between ferroptosis and mitophagy components interferes with mitochondrial homeostasis. Inhibition of O-GlcNAcylation leads to mitochondrial fragmentation and enhanced mitophagy, providing additional sources of labile iron and rendering the cell more sensitive to ferroptosis [129]. Inhibition of Complex I by BAY 87-2243 (BAY) depolarized the mitochondrial membrane potential (Δψ), increased cellular ROS levels, stimulated lipid peroxidation and reduced glutathione levels, thus leading to ferroptosis of melanoma cells (Fig. 4). This process also opens the mitochondrial permeability transition pore (mPTP) and enhances the stimulation of autophagosome formation and mitophagy. Knockdown of autophagy-related 5 (ATG5) inhibited the BAY-stimulated autophagosome formation, cellular ROS increase and ferroptosis [130]. In Type 2 diabetic osteoporosis, mitochondrial ferritin deficiency promotes osteoblastic ferroptosis via mitophagy [131].

## Targeting ferroptosis as a vulnerability in cancer cells via mitochondria

Cell death mediated via ferroptosis shows potential in cancer treatment. In recent years, increasing evidence has indicated ferroptosis-based vulnerability in cancer cells [132]. Given the importance of mitochondria in ROS production and ferroptosis regulation, we will focus on mitochondrial ferroptosis-related tumor inhibition and exploit their potential as cancer therapies.

Similar to extramitochondrial ferroptotic regulatory pathways, the ferroptosis regulatory pathways mediated via the mitochondria are classified into GPX4-dependent (mGPX4) and GPX4-independent (DHODH-COQH_2_ axis) pathways. GPX4 drives the most powerful defense against ferroptosis and some drug-resistant/tolerant cancer cells are considerably vulnerable to GPX4-mediated ferroptosis [20, 21]. No drug specifically targets mGPX4, but the feasibility of developing general GPX4 inhibitors for cancer therapy has been verified with many cancer models, including melanoma [133], renal cell carcinoma [134], small cell lung cancer [135], breast cancer [136–139], and other models [20, 140].

The mGPX4- and DHODH- centered axes are usually complementary and the inactivation of one generally increases the reliance on the other. Overexpression of mGPX4 in cancer cells increases their resistance to DHODH-inhibition-induced lipid peroxidation and ferroptosis. Interestingly, cGPX4 restoration is not sufficient to demonstrate this complementary relationship [50]. Combined inactivation of both mitochondrial pathways strongly induces mitochondrial dysfunction and ferroptosis. Therefore, DHODH may be a combinative therapeutic target that induces ferroptosis-mediated cell death in GPX4^low^ cancers.

However, recent findings have suggested that targeting DHODH alone may be sufficient to induce ferroptosis under certain circumstances. Zhang et.al reported that DHODH inhibition alone was sufficient to induce lipid peroxidation and ferroptosis in tumor cells [141]. The authors prove that the manganese (Mn^2+^) treatment-induced tumor cell death is irrelevant to the apoptosis, necroptosis, pyroptosis or autophagy, but only to ferroptosis, in which type I interferons (IFNs) were the main mediators of downregulated DHODH expression and subsequent mitochondrial ROS accumulation; however, the expression of components in other ferroptosis pathways, such as the GPX4, FSP1-CoQ10, and GCH1-BH4 pathways, remained unchanged [141]. Moreover, Mn^2+^-initiated ferroptosis was attenuated with supplementation with DHO or Type I IFN inhibitors, which restored the expression of DHODH [141]. Additionally, another group demonstrated that benzene-induced anemia is involved only in DHODH-mediated ferroptosis at least in the bone marrow [142]. Benzene treatment of B lymphocytes significantly decreased the expression of DHODH and increased ferroptosis in vitro without changing the expression of GPX4 or FSP1 [142].

Although CoQ has been detected in nonmitochondrial membranes, CoQ is synthesized mainly in mitochondria [143]. CoQ is involved not only in DHODH axis but also in the FSP-CoQ10 axis activation. Exogenous CoQ supplementation protected membrane lipids from peroxidation, maintained mitochondria functionality, and increased cell resistance to ferroptotic stimuli [144]. This raises the question of how mitochondrial metabolism influences ferroptosis. Mitochondria, the major sources of ROS production, are necessary for lipid peroxidation and ferroptosis [145]. A mitochondrion is a cellular energy manufacturer that provides ATP for ferroptosis-related enzyme activity. Electron transport and proton pumping suppression in mitochondria can inhibit ferroptosis induction [99]. Hence, mitochondria are extremely susceptible to lipid peroxidation and ferroptosis [146]. Although few studies have been reported, targeting the vulnerability of mitochondrial ferroptosis for cancer therapy will undoubtfully be a promising and exciting treatment in the near future.

## Conclusion and perspectives

The role of mitochondria in RCD including apoptosis, necrosis, pyroptosis and ferroptosis has been studied in recent years. Cuproptosis discovered in 2022 is called a copper-triggered modality of mitochondrial cell death [147, 148]. We summarized its role in ferroptosis in the above four parts. Ferroptosis is a form of RCD caused by iron-dependent peroxidation of polyunsaturated phospholipids on cell membranes and is actively suppressed by the cellular antioxidant systems. Ferroptosis is controlled by integrated oxidation and antioxidant systems and ROS are the oxidants. Irreversible damage to mitochondria function and integrity caused by excessive oxidative stress leads to energy depletion and ferroptosis.

Mitochondria-deficient cells were still sensitive to a panel of ferroptosis-inducing compounds and the cytoactive was rescued by ferrostatins and iron chelators in another research. Considering the pivotal role of mitochondria in tumor cell metabolic rewiring, it is possible that modulation of the mitochondrial metabolic pathways might reshape the tumor microenvironment, thus leading to ferroptosis-mediated tumor suppression. Besides mitochondria, other organelles also have close relations with ferroptosis, for example, lysosomes also play an important role in iron metabolism. Small amounts of uncoordinated and redox-active Fe^2+^ form the “labile iron pool” in cells and lysosomes recycle endogenous iron sources like ferritin and mitochondria, thus lysosome is also a particularly large “liable iron pool” [149, 150].

Characteristics and biological significance of ferroptosis are studied broadly and fast. Targeting or inducing the ferroptosis of cancer cells has been treated as a novel therapeutic method [151, 152]. In the MYCN-amplified neuroblastoma, the iron flux was increased and the growth of cancer cells is highly reliable to the system Xc^−^/Glutathione Axis [153]. In prostate cancer, ferropotosis inducers such as erastin and RSL3 inhibit cancer cell growth [154]. In triple-negative breast cancer, its inhibitor DMOCPTL induced ferroptosis of cancer cells by the ubiquitination of GPX4 of via regulating the activity of EGR1 [155]. In addition, stem cells orchestrate ferroptosis to keep their growth, e.g. stem cell factor SOX2 confers ferroptosis resistance in lung cancer via upregulation of SLC7A11 [5, 156]. Therefore, induction ferroptosis of stem cells would be a promising method to clear cancer stem cells.

The roles of mitochondria in ferroptosis are being explored. Intriguing questions need to be answered to increase our understanding: (1) Targeting mitochondria is a novel cancer treatment, but how can cancer cell mitochondria be selectively targeted to induce ferroptosis? (2) How can cells undergoing ferroptosis be rescued by targeting mitochondria? (3) Does mitochondrial metabolism reprogramming function in simultaneous conjunction with ferroptosis? Answers to these and other questions will further illustrate the roles of mitochondria in ferroptosis and the application of ferroptosis t cancer treatment.

## Data Availability

All data generated or analyzed during this study are included in this published article.
